# Differential Immune Checkpoint Expression in CD4^+^ and CD4^−^ NKT Cell Populations During Healthy Pregnancy

**DOI:** 10.3390/ijms26168022

**Published:** 2025-08-19

**Authors:** Matyas Meggyes, Nagy U. David, Livia Mezosi, Fanni Vastag, Dora Kevey, Laszlo Szereday

**Affiliations:** 1 Medical School, Department of Medical Microbiology, University of Pecs, 7624 Pecs, Hungary; livimezosi@gmail.com (L.M.); szereday.laszlo@pte.hu (L.S.); 2 Institute of Geobotany/Plant Ecology, Martin-Luther-University, Große Steinstraße 79/80, D-06108 Halle (Saale), Germany; davenagy9@gmail.com; 3 Medical School, Department of Obstetrics and Gynaecology, University of Pecs, 7624 Pecs, Hungary; vfanny92@gmail.com (F.V.); keveydorka@gmail.com (D.K.)

**Keywords:** NKT cells, CD4, immune checkpoint pathways, pregnancy, trimester

## Abstract

This study investigated the expression of immune checkpoint molecules on CD4^+^ and CD4^−^ NKT cell subpopulations throughout healthy pregnancy trimesters and in non-pregnant condition to understand their role in maternal–fetal immunotolerance. Using flow cytometry, we found that CD4^−^ NKT cells significantly outnumbered CD4^+^ NKT cells in all investigated groups. In the case of the immune checkpoint molecules, PD-1 receptor expression was significantly lower in CD4^−^ NKT cells compared to CD4^+^ counterpart cells only in non-pregnant women, while the PD-L1 ligand expression on CD4^+^ NKT cells significantly decreased in the third trimester. In contrast, LAG-3 and Galectin-3 expressions remained stable across all subsets and trimesters. For the TIGIT/CD226 axis, CD226 expression was significantly higher in CD4^+^ NKT cells in the third trimester and in non-pregnant women. The two ligands CD112 and CD155 were consistently lower on CD4^−^ NKT cells across all groups. The activating receptor NKG2D was significantly higher on CD4^−^ NKT cells in all examined cohorts. These findings suggest that CD4^+^ NKT cells tend towards a more tolerogenic phenotype, while CD4^−^ NKT cells maintain a balanced cytotoxic potential with reduced immunoregulation function. The dynamic regulation of immune checkpoints on NKT cell subsets, particularly the downregulation of PD-L1 and CD226 in late pregnancy, highlights their fine-tuned role in balancing maternal–fetal immune tolerance with readiness for parturition.

## 1. Introduction

Natural killer T (NKT) cells are a unique subset of T lymphocytes that function as a bridge between the innate and adaptive immune systems. A major NKT cell subset is restricted by the MHC class I-like molecule CD1d [1], which is expressed on cells in the bone marrow, thymus, and liver and is also found in the spleen and other peripheral lymphoid organs [2]. These cells express diverse T-cell receptors (TCRs) with broader lipid antigen specificities; however, they remain difficult to identify, and little is known about their development. Based on expression of T-cell lineage markers CD4 and CD8, NKT cells can be subdivided into three populations: CD4^+^CD8^−^ (CD4^+^), CD4^−^CD8^−^ (double-negative (DN)) and CD4^−^CD8^+^ (CD8^+^). Unlike conventional CD8αβ^+^ T cells, but similar to NK and γδ T cells, NKT cells express CD8 exclusively as the homodimer CD8αα [3].

Phenotypic analysis of CD3^+^CD56^+^ T cells reveals that they are primarily composed of CD8+ and γδTCR+ T cells (36.1% and 27.1%, respectively), whereas CD4^+^, DN and MAIT cells represent smaller fractions (6.4%, 9%, and 4.9%, respectively). The biological function of NKT cells is multifaceted and context-dependent, as they can rapidly produce large amounts of both T helper 1 (Th1) and Th2 cytokines, supporting or suppressing cell-mediated immunity depending on the setting [4]. CD4^+^ NKT cells produce both Th1- and Th2-type cytokines (e.g., IFN-γ, TNF, IL-4, IL-10, IL-13), while DN NKT cells are biased toward Th1-type cytokines (IFN-γ and TNF) [4,5,6]. Expression of cytolytic enzymes aligns with a central memory-like phenotype, with most NKT cells expressing granzyme A, but only a few expressing granzyme B, and virtually none expressing perforin [3]. This positions NKT cells within a unique immunological niche resembling central memory T cells.

Immune checkpoint molecules serve as crucial modulators of immune responses, acting as regulatory “brakes” to prevent immune overactivation and ensure self-tolerance. These molecules are typically expressed on effector immune cells such as T and NK cells, where they interact with ligands on antigen-presenting or tissue cells to deliver inhibitory or stimulatory signals that fine-tune immune responses [7]. While extensive research has been devoted to immune checkpoint inhibitors in cancer immunotherapy [8,9], reproductive immunology remains a comparatively underexplored and sometimes controversial domain [8].

Among these pathways, the PD-1/PD-L1 axis plays a crucial role in establishing and maintaining maternal–fetal tolerance during pregnancy [9]. The semi-allogeneic fetus expresses paternal antigens that could otherwise provoke maternal immune rejection. PD-L1 is highly expressed on placental trophoblasts, creating a local immunosuppressive environment that protects fetal tissues from immune attack [10]. PD-1 expression on maternal immune cells (e.g., T cells) allows this checkpoint to deliver inhibitory signals upon PD-L1 engagement, suppressing T-cell activation [11]. Beyond suppressing immune activation, the PD-1/PD-L1 interaction also supports the differentiation and function of regulatory T cells (Tregs), which are vital for maintaining tolerance at the maternal–fetal interface [11,12]. Dysregulation of this pathway has been associated with complications such as recurrent spontaneous abortion (RSA) and preeclampsia, highlighting its clinical significance [11]. Understanding the precise role of this pathway in these conditions may offer potential therapeutic targets for intervention.

Similar to PD-1, the LAG-3 (Lymphocyte Activation Gene-3)/Galectin-3 (Gal-3) pathway contributes to maternal–fetal tolerance by suppressing excessive maternal immune activation [9]. Engagement of LAG-3 by Gal-3 leads to reduced T cell proliferation, diminished cytokine production, and potential Treg induction [13]. Gal-3 is expressed by placental trophoblast and decidua cells, enabling interaction with LAG-3+ immune cells infiltrating the maternal–fetal interface [14]. Soluble forms of LAG-3 and Gal-3 found in the maternal circulation may also exert systemic immunomodulatory effects during pregnancy [15]. While this pathway is essential for fetal tolerance, tumors can evade immune surveillance, prompting the development of LAG-3 inhibitors for cancer immunotherapy. This raises important questions about the safety of such therapies during pregnancy.

The TIGIT (T-cell immunoreceptor with Ig and ITIM domains)/CD226 (DNAX accessory molecule-1) axis, along with its shared ligands CD112 (nectin-2) and CD155 (poliovirus receptor), constitutes another vital checkpoint pathway at the maternal–fetal interface [16]. TIGIT binding to CD112 or CD155 delivers inhibitory signals, suppressing T-cell proliferation and cytokine production while enhancing Treg function [17]. In contrast, CD226 engagement by the same ligands promotes immune activation, underscoring the importance of tightly regulated expression of TIGIT and CD226 during pregnancy [18,19]. CD112 and CD155, which are abundantly expressed on antigen-presenting and trophoblast cells, interact with TIGIT and CD226 on effector immune cells (e.g., T and NK cells), thereby shaping local immune responses [20]. The balance between these inhibitory and activating signals likely plays a crucial role in ensuring fetal tolerance without compromising immune surveillance.

In summary, immune checkpoint molecules play a central role in preserving immune balance during pregnancy. Investigating their expression on NKT cell subpopulations—especially CD4^+^ and CD4^−^ subsets—across different trimesters may provide critical insights into the mechanisms of maternal–fetal immunotolerance.

## 2. Results

### 2.1. The Frequency of CD4^+^ and CD4^−^ NKT Cell Subpopulations in Healthy Pregnancy and Non-Pregnant Women

**Figure 1:**
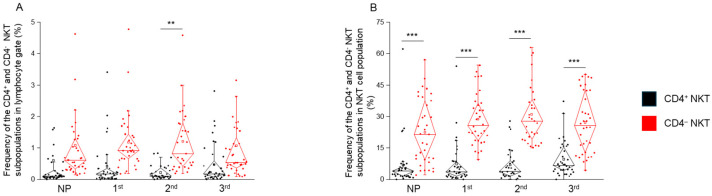
**Frequency of CD4^+^ and CD4^−^ NKT cells in peripheral blood across pregnancy trimesters and non-pregnant controls.** The frequency of the CD4^+^ and CD4^−^ NKT cells in the lymphogate (**A**) and in the total NKT population (**B**) in the three trimesters of healthy pregnancy and non-pregnant women. The solid bars represent medians of 35, 34, 31 and 36 determinations, respectively; the boxes indicate the interquartile ranges; and the whiskers represent the lower and upper 25% of the data between the minimum and maximum values. At the same time, the outliers are values that are at least one and a half times smaller or larger than the interquartile range from the lower or upper quartile. Statistically significant differences with *p*-values < 0.01 *** and <0.03 ** are indicated. NP: non-pregnant, 1st: first trimester, 2nd: second trimester, 3rd: third trimester.

CD4^+^ and CD4^−^ NKT subpopulations were identified using multicolor flow cytometry. Based on our analysis, these two subsets were distinguishable by CD4 expression. A comparison of their distribution revealed significant differences across all study groups. When assessed within the lymphocyte gate, the frequency of CD4^−^ NKT cells was significantly higher than that of CD4^+^ NKT cells specifically during the second trimester of pregnancy (Figure 1A). Furthermore, when frequencies were calculated relative to the total NKT cell population (Figure 1B), the frequency of CD4^−^ NKT cells was significantly higher compared to CD4^+^ NKT cells in all three trimesters of pregnancy as well as in the non-pregnant state. When comparing each subset to the non-pregnant controls, no trimester-dependent differences were observed for CD4^+^ or CD4^−^ NKT frequencies, whether expressed within the lymphocyte gate or relative to total NKT (Figure 1A,B).

### 2.2. PD-1 and PD-L1 Expression by CD4^+^ and CD4^−^ NKT Cell Subpopulations During Healthy Pregnancy and in Non-Pregnant Women

**Figure 2:**
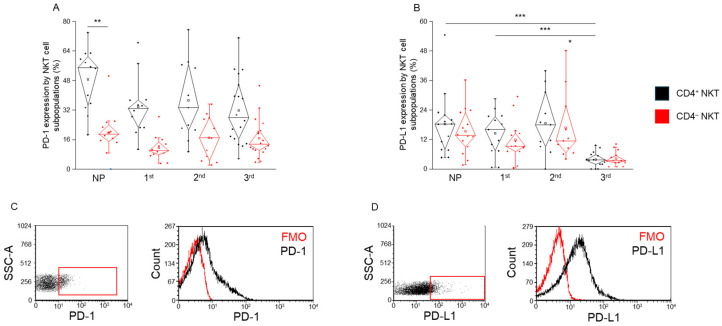
**PD-1 and PD-L1 expression by CD4^+^ and CD4^−^ NKT subpopulations during healthy pregnancy and non-pregnant controls.** PD-1 receptor expression (**A**) and PD-L1 ligand expression (**B**) by the CD4^+^ and CD4^−^ NKT subpopulations in the three trimesters of healthy pregnancy and in non-pregnant women. The solid bars represent medians of 14, 13, 11, and 18, determinations respectively; the boxes indicate the interquartile ranges; and the whiskers represent the lower and upper 25% of the data between the minimum and maximum values, while the outliers are values that are at least one and a half times smaller or larger than the interquartile range from the lower or upper quartile. Statistically significant differences with *p*-values <0.01 ***, <0.03 ** and <0.05 * are indicated. Representative FACS plots show the PD-1 surface marker (**C**) and PD-L1 surface molecule (**D**) expression by cells in the lymphocyte gate. To determine the positivity of PD-1 and PD-L1, a fluorescent minus one (FMO) control was used. NP: non-pregnant, 1st: first trimester, 2nd: second trimester, 3rd: third trimester.

PD-1 expression was significantly lower in CD4^−^ NKT cells compared to CD4^+^ NKT cells in the non-pregnant group (Figure 2A). Although this trend of reduced PD-1 expression in CD4^−^ subset was also observed in all pregnancy trimesters, the differences did not reach statistical significance within these cohorts (Figure 2A).

Regarding PD-L1 expression, a significant reduction was observed in CD4^+^ NKT cells from women in the third trimester compared to both the non-pregnant group and the first-trimester group (Figure 2B). This suggests a trimester-specific downregulation of PD-L1 in CD4^+^ NKT cells as pregnancy progresses.

### 2.3. LAG-3 and Gal-3 Expression by CD4^+^ and CD4^−^ NKT Cell Subpopulations During Healthy Pregnancy and in Non-Pregnant Women

**Figure 3:**
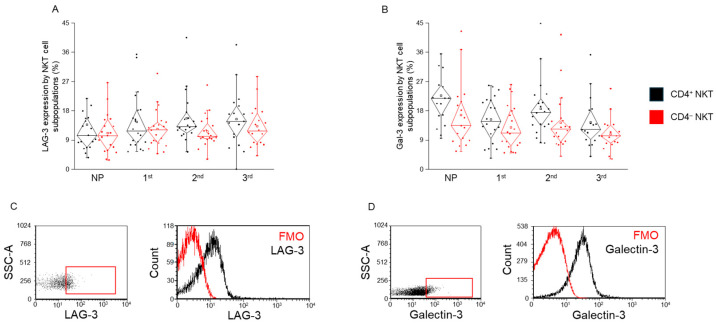
**LAG-3 and Gal-3 expression by CD4^+^ and CD4^−^ NKT subpopulations during healthy pregnancy and non-pregnant controls.** LAG-3 receptor expression (**A**) and Gal-3 ligand expression (**B**) by the CD4^+^ and CD4^−^ NKT subpopulations in the three trimesters of healthy pregnancy and in non-pregnant women. The solid bars represent medians of 19, 20, 19 and 18 determinations, respectively; the boxes indicate the interquartile ranges; and the whiskers represent the lower and upper 25% of the data between the minimum and maximum values, while the outliers are values that are at least one and a half times smaller or larger than the interquartile range from the lower or upper quartile. Representative FACS plots show the LAG-3 surface marker (**C**) and Gal-3 molecule (**D**) expression by cells in the lymphocyte gate. To determine the positivity of LAG-3 and Gal-3, FMO control was used. NP: non-pregnant, 1st: first trimester, 2nd: second trimester, 3rd: third trimester.

No significant differences in LAG-3 receptor or Gal-3 ligand expression were observed between CD4^+^ and CD4^−^ NKT cell subpopulations in any of the groups analyzed (Figure 3). Furthermore, when comparing expression levels across different trimesters of pregnancy and the non-pregnant control group, no statistically significant differences were detected for either molecule (Figure 3). These findings suggest that LAG-3 and Gal-3 expression remains stable across NKT subsets and pregnancy stages under healthy conditions.

### 2.4. TIGIT and CD226 Receptor Expression by CD4^+^ and CD4^−^ NKT Cell Subpopulations During Healthy Pregnancy and in Non-Pregnant Women

**Figure 4:**
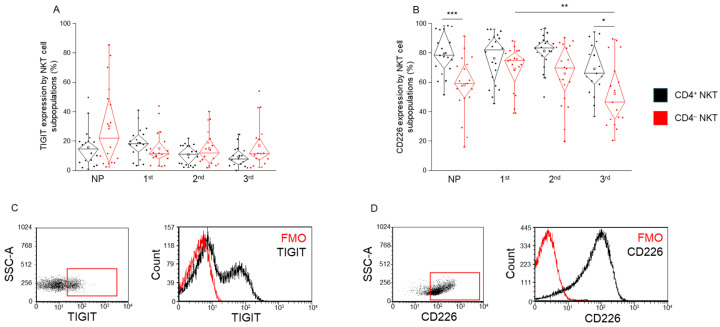
**TIGIT and CD226 expression by CD4^+^ and CD4^−^ NKT subpopulations during healthy pregnancy and non-pregnant controls.** TIGIT receptor expression (**A**) and CD226 receptor expression (**B**) by the CD4^+^ and CD4^−^ NKT subpopulations in the three trimesters of healthy pregnancy and in non-pregnant women. The solid bars represent medians of 19, 20, 20, and 17 determinations, respectively; the boxes indicate the interquartile ranges; and the whiskers represent the lower and upper 25% of the data between the minimum and maximum values, while the outliers are values that are at least one and a half times smaller or larger than the interquartile range from the lower or upper quartile. Statistically significant differences with *p*-values <0.01 ***, <0.03 ** and <0.05 * are indicated. Representative FACS plots show the TIGIT surface marker (**C**) and CD226 surface molecule (**D**) expression by cells in the lymphocyte gate. To determine the positivity of TIGIT and CD226, FMO control was used. NP: non-pregnant, 1st: first trimester, 2nd: second trimester, 3rd: third trimester.

No significant differences in TIGIT receptor expression were observed between CD4^+^ and CD4^−^ NKT cell subpopulations in any of the groups analyzed (Figure 4A). In contrast, CD226 expression was significantly higher in CD4^+^ NKT cells compared to the CD4^−^ NKT cells in the non-pregnant group and the third-trimester group (Figure 4B). Additionally, a significant decrease in CD226 expression was observed in the third trimester compared to the first trimester, indicating a downregulation of the activatory receptor as pregnancy progresses, but this effect was limited to the CD4^−^ subset (Figure 4B).

### 2.5. TIGIT and CD226 MFI and the Frequency of Double-Positive NKT Cell Subpopulations in Healthy Pregnancy and Non-Pregnant Women

**Figure 5:**
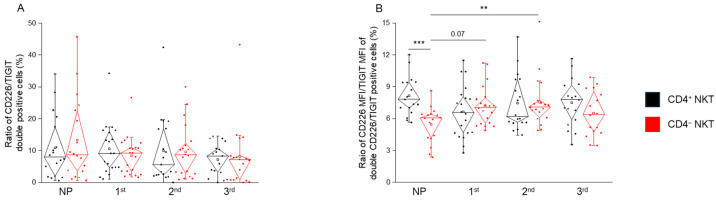
**Ratio of TIGIT/CD226 double-positive cells and the ratio of CD226 MFI/TIGIT MFI by different NKT cells during healthy pregnancy and non-pregnant controls**. Frequency of the TIGIT/CD226 double-positive NKT cell subpopulations (**A**) and the ratio of the CD226 MFI/TIGIT MFI by the TIGIT/CD226 double-positive NKT cell subpopulation (**B**) receptors in the three trimesters of healthy pregnancy and non-pregnant women. The solid bars represent medians of 17, 20, 18, and 17 determinations, respectively; the boxes indicate the interquartile ranges; and the whiskers show the most extreme observations. The middle square within the box represents the mean value. Statistically significant differences with *p*-values <0.01 *** and <0.03 ** are indicated. NP: non-pregnant, 1st: first trimester, 2nd: second trimester, 3rd: third trimester.

No significant difference was found in the ratio of the TIGIT/CD226 double-positive NKT cell subpopulations across any of the groups examined (Figure 5A). However, the ratio of the CD226 MFI value and the TIGIT MFI value of these double-positive cells was significantly lower in CD4^−^ NKT cells compared to the CD4^+^ counterpart, but only in the non-pregnant group (Figure 5B). Additionally, in CD4^−^ NKT cells, this MFI ratio was significantly higher in the second-trimester group compared to the non-pregnant group, suggesting a pregnancy-associated modulation of receptor expression intensity specifically within this subset (Figure 5B).

### 2.6. CD112 and CD155 Ligand Expression by CD4^+^ and CD4^−^ NKT Cell Subpopulations in Healthy Pregnancy and Non-Pregnant Women

**Figure 6:**
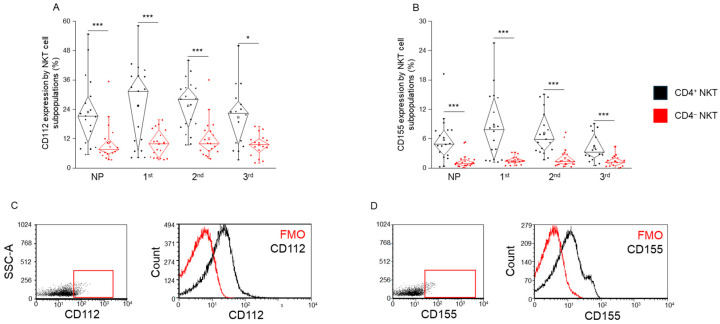
**CD112 and CD155 expression by CD4^+^ and CD4^−^ NKT subpopulations during healthy pregnancy and non-pregnant controls.** CD112 receptor expression (**A**) and CD155 receptor expression (**B**) by the CD4^+^ and CD4^−^ NKT subpopulations in the three trimesters of healthy pregnancy and non-pregnant women. The solid bars represent medians of 18, 19, 19, and 16 determinations, respectively; the boxes indicate the interquartile ranges; and the whiskers represent the lower and upper 25% of the data between the minimum and maximum values, while the outliers are values that are at least one and a half times smaller or larger than the interquartile range from the lower or upper quartile. Statistically significant differences with *p*-values <0.01 ***, and <0.05 * are indicated. Representative FACS plots show the CD112 surface marker (**C**) and CD155 surface molecule (**D**) expression by cells in the lymphocyte gate. To determine the positivity of CD112 and CD155, FMO control was used. NP: non-pregnant, 1st: first trimester, 2nd: second trimester, 3rd: third trimester.

Surface expression of the CD112 ligand was significantly lower in CD4^−^ NKT cells compared to CD4^+^ NKT cells across all groups, including all trimesters of pregnancy and the non-pregnant controls (Figure 6A). A similar expression pattern was observed for CD155, with CD4^−^ NKT cells consistently exhibiting significantly lower surface levels of this ligand compared to their CD4^+^ counterparts in every group analyzed (Figure 6B).

These findings indicate a subset-specific expression profile of checkpoint ligands, with CD4^−^ NKT cells expressing lower levels of both CD112 and CD155, regardless of pregnancy status.

### 2.7. Expression of the Activation Receptor NKG2D by CD4^+^ and CD4^−^ NKT Cell Subpopulations During Healthy Pregnancy and in Non-Pregnant Women

**Figure 7:**
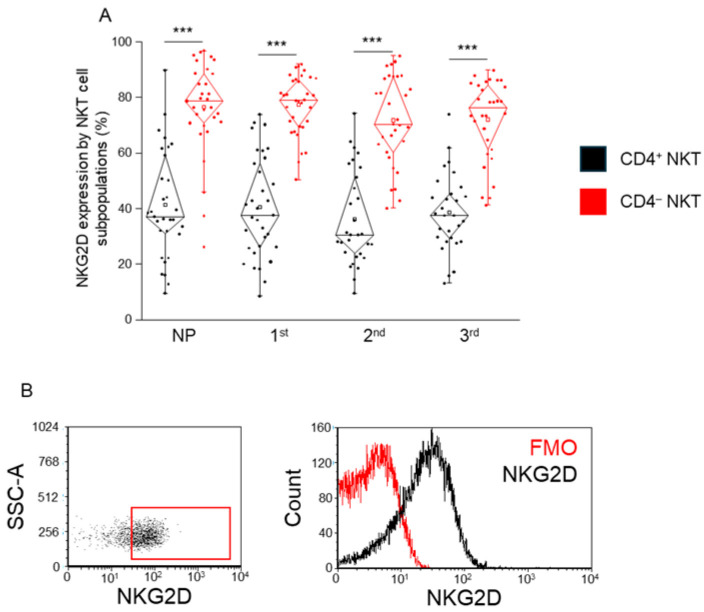
**NKG2D expression by CD4^+^ and CD4^−^ NKT subpopulations during healthy pregnancy and in non-pregnant controls.** NKG2D receptor expression (**A**) by the CD4^+^ and CD4^−^ NKT subpopulations in the three trimesters of healthy pregnancy and in non-pregnant women. The solid bars represent medians of 31, 34, 31, and 30 determinations, respectively; the boxes indicate the interquartile ranges; and the whiskers represent the lower and upper 25% of the data between the minimum and maximum values, while the outliers are values that are at least one and a half times smaller or larger than the interquartile range from the lower or upper quartile. Statistically significant differences with *p*-values <0.01 *** are indicated. Representative FACS plots show the NKG2D surface marker (**B**) expression by cells in the lymphocyte gate. To determine the positivity of NKG2D, FMO control was used. NP: non-pregnant, 1st: first trimester, 2nd: second trimester, 3rd: third trimester.

NKG2D expression was significantly higher in CD4^−^ NKT cells compared to CD4^+^ NKT cells across all cohorts analyzed, including each trimester of pregnancy and the non-pregnant control group (Figure 7A).

## 3. Discussion

A healthy pregnancy requires substantial adaptations in the maternal immune system. The immunological trajectory of pregnancy, shifting from a pro-inflammatory milieu in the first trimester to tolerance in the second and reverting to inflammation in preparation for labor, necessitates dynamic regulation of effector cell populations [21]. In this context, NKT cells may serve as rapid responders balancing surveillance and tolerance, yet their precise regulatory landscape during gestation remains understudied. In response to the presence of the fetus, the maternal immune system adapts to ensure the fetus’s undisturbed development, while still preserving antimicrobial and antitumor immunity. This immunological adjustment, known as maternal immune tolerance, involves a delicate balance between pro-inflammatory and anti-inflammatory responses, and its disruption can lead to complications affecting both the mother and the fetus.

Immune checkpoint molecules are key regulators of this balance. Through receptor–ligand interactions, these molecules mediate co-stimulatory or co-inhibitory signals that can shape immune responses toward tolerance or inflammation. Numerous studies, including our own, have investigated the potential roles of IC molecules in the establishment and maintenance of maternal immune tolerance during pregnancy [8,11,22,23,24,25,26]. While most prior research has focused on effector cell populations such as T and NK cells, no previous study has examined NKT cells based on CD4 expression across gestational stages.

Classical NKT cells are a distinct lymphocyte population that bridges the innate and adaptive immune systems, and they are capable of rapid cytokine production and immune modulation upon encountering an antigen. This functional versatility enables them to participate in pathogen defense, tumor surveillance, allergic reactions, and, potentially, maternal–fetal immune tolerance [27]. Previous studies in both human and murine models have shown that CD4^+^ and double-negative (CD4^−^/CD8^−^) NKT cells originate from distinct developmental lineages and exhibit functionally distinct profiles [3,28,29]. Our findings confirm that the CD4^−^ NKT subpopulation significantly outnumbers the CD4^+^ subset across all stages of pregnancy and in non-pregnant controls. While this ratio remained stable throughout pregnancy, the dominance of CD4^−^ cells is nonetheless noteworthy. This stable distribution suggests a potentially conserved immunological role for CD4^−^ NKT cells in maintaining maternal immune balance. Importantly, CD4^+^ NKT cells are known to secrete high levels of IL-2 following activation by dendritic cells and α-galactosylceramide, a response that may promote Treg expansion [30]. Given the essential role of Tregs in establishing and maintaining maternal immune tolerance, the presence and activity of CD4^+^ NKT cells may indirectly support a tolerogenic environment during gestation, even if they represent a numerically smaller subset.

Building on the phenotypic distinction between CD4^+^ and CD4^−^ NKT cells, our data revealed important differences in immune checkpoint molecule expression that may reflect functional specialization within these subsets. The opposing expression patterns of inhibitory (PD-1, TIGIT) and activatory (CD226, NKG2D) receptors between CD4^+^ and CD4^−^ subsets likely reflect a checkpoint-mediated specialization, with CD4^+^ cells supporting tolerance and CD4^−^ cells preserving immune surveillance capacity.

PD-1 expression was significantly higher in CD4^+^ NKT cells than in CD4^−^ counterparts, but only in the non-pregnant group, suggesting a baseline predisposition of CD4^+^ cells toward a more regulated or exhaustion-prone phenotype. While the same trend persisted throughout pregnancy, no significant differences were found between the subsets in pregnant women, possibly reflecting pregnancy-induced modulation of PD-1 regulation. Interestingly, PD-L1 expression was significantly lower on CD4^+^ NKT cells in the third trimester, compared to earlier trimesters and the non-pregnant group. The reduced PD-L1 expression in CD4^+^ NKT cells during the third trimester coincides with the immunological reversion toward pro-inflammatory readiness preceding parturition. This may reflect the dismantling of immune suppression at the maternal–fetal interface in preparation for delivery. This reduction may suggest a late pregnancy shift away from active immune suppression, possibly preparing the maternal immune system for parturition.

In contrast, LAG-3 and Galectin-3 expression remained unchanged across all subpopulations and gestational stages, indicating that this checkpoint pathway may not be differentially regulated in NKT cells during healthy pregnancy. The static expression of LAG-3 and Gal-3 suggests that these molecules may play a constitutive rather than regulatory role in NKT cells, or that their influence is eclipsed by more dynamic pathways such as PD-1 or TIGIT during pregnancy.

The TIGIT/CD226 axis, which balances inhibitory and activatory signals, showed more dynamic behavior. While TIGIT expression did not differ significantly between CD4^+^ and CD4^−^ NKT cells, CD226 expression was consistently higher in CD4^+^ NKT cells, especially in the non-pregnant and third-trimester groups. Furthermore, CD226 expression in CD4^−^ NKT cells decreased significantly in the third trimester compared to the first trimester, suggesting a subset-specific dampening of activation potential as pregnancy progresses. This subset-specific modulation may signify a functional polarization of CD4^−^ NKT cells away from an activatory phenotype, aligning with a broader gestational need for immune dampening. Given the role of CD226 in promoting IFN-γ production and cytotoxicity, its downregulation may serve as a brake to prevent fetal rejection.

These findings were further supported by MFI ratio analysis of CD226 to TIGIT in double-positive cells, where CD4^−^ NKT cells in the non-pregnant group exhibited significantly lower ratios than CD4^+^ cells, indicating a relatively inhibitory receptor bias. This ratio was significantly elevated in the second trimester within the CD4^−^ subset, perhaps reflecting a temporary shift toward a more balanced or activatory immune profile during mid-pregnancy.

Moreover, the ligands CD112 and CD155, shared by TIGIT and CD226, were consistently lower in CD4^−^ NKT cells, regardless of gestational stage. The consistently lower ligand expression on CD4^−^ NKT cells may reflect a cell-intrinsic dampening of checkpoint signaling capacity, perhaps limiting their activation in peripheral immune surveillance to avoid collateral tissue damage during gestation. This finding highlights a reduced capacity for checkpoint engagement by CD4^−^ cells, which may influence their activation thresholds and interactions with antigen-presenting cells.

Interestingly, the activating receptor NKG2D was significantly more expressed on CD4^−^ NKT cells in all groups, indicating that this subset may retain a stronger cytotoxic potential. This contrasts with their lower expression of co-stimulatory and co-inhibitory checkpoint molecules, and may imply a less tightly regulated effector state, possibly contributing to early immunosurveillance or inflammatory responses if dysregulated. The elevated NKG2D expression in CD4^−^ NKT cells, despite their lower checkpoint receptor levels, suggests a poised cytotoxic readiness, potentially important for rapid pathogen or danger signal response without requiring classical TCR stimulation.

Complementing our prior analysis of CD8^+^ vs. CD8^−^ NKT cells [24]—where CD8^+^ NKT showed elevated TIGIT, increased CD155 in the third trimester, a reduced CD226/TIGIT ratio, and lower PD-L1 in the third trimester—our present study, restricted to CD8^−^ NKT and stratified by CD4, reveals a more selective pattern: a third-trimester PD-L1 decrease on CD4^+^ NKT with otherwise minimal CD4^−^-dependent divergence. Together, these data indicate that CD8 status exerts the stronger influence on NKT checkpoint configuration during healthy pregnancy, whereas CD4 status within CD8^−^ NKT cells fine-tunes PD-L1-mediated regulation.

This study is limited by its cross-sectional design, which does not allow for longitudinal tracking of immune changes within individuals across trimesters. Additionally, functional assays were not performed to directly assess the cytokine secretion or cytotoxic activity of NKT subpopulations, which would further clarify the biological relevance of the observed phenotypic differences. Future studies should integrate functional assays, including cytokine profiling and cytotoxicity assessment, to contextualize the phenotypic observations. Understanding these dynamics could unveil new biomarkers or therapeutic targets for pregnancy complications associated with immune dysregulation, such as preeclampsia or miscarriage.

Taken together, our findings support a model in which CD4^+^ and CD4^−^ NKT cell subsets make different contributions to maternal–fetal immune regulation. CD4^+^ NKT cells appear to adopt a more regulated or tolerogenic phenotype, characterized by higher expression of PD-1 and CD226, which potentially favors interaction with antigen-presenting cells and promotes Treg activity. In contrast, CD4^−^ NKT cells exhibit higher expression of NKG2D and lower levels of checkpoint ligands (CD112, CD155), indicating a poised cytotoxic phenotype with reduced immunoregulation, which may facilitate rapid surveillance functions. The downregulation of PD-L1 and CD226 in late pregnancy may reflect a shift toward inflammatory readiness in preparation for labor and delivery. These subset-specific and trimester-dependent expression profiles suggest that the NKT compartment is finely tuned to balance tolerance and immune competence at the maternal–fetal interface.

## 4. Materials and Methods

### 4.1. Ethical Approval

All participants provided written informed consent, adhering to a protocol approved by the Medical School, University of Pecs, Regional and Local Research Ethics Committee (Reference: 6149). The research was conducted in accordance with the ethical principles outlined in the 1975 Declaration of Helsinki.

### 4.2. Participants

Pregnant participants were recruited from the Department of Obstetrics and Gynaecology at the University of Pecs. The cohort included 32 women in the first trimester, 29 in the second trimester, and 35 in the third trimester (Table 1). A control group of 33 healthy, age-matched, non-pregnant women was included for comparison (Table 1).

To comply with EU-GDPR and data protection regulations, all personal and health information was processed anonymously and securely, preventing demographic analysis by the research team.

All participants underwent health screening. Exclusion criteria included significant medical history, current medication use, acute or chronic illness, pregnancy-related complications, pre-existing conditions, IVF pregnancies, immune-related disorders, diabetes mellitus, or HIV/AIDS. Only healthy and spontaneously conceived pregnancies were included.

### 4.3. Sample Collection, PBMC Separation, and Cryopreservation

A total of 20 mL of venous blood was collected from each participant using heparinized tubes and immediately transported to the laboratory for processing. Peripheral blood mononuclear cells (PBMCs) were isolated using a Ficoll–Paque density gradient (GE Healthcare) centrifugation method. Following isolation, the cells were washed twice in RPMI 1640 medium (Lonza), counted, and then centrifuged.

For long-term storage, PBMCs were resuspended in human serum containing 10% DMSO (Sigma-Aldrich, Budapest, Hungary) and aliquoted into cryovials. Samples were then stored at −80 °C until further analyses. Prior to fluorescent antibody labeling, PBMCs were rapidly thawed in a 37 °C water bath, and DMSO was removed by two washes with RPMI 1640 medium.

### 4.4. Flow Cytometric Measurement

Thawed PBMCs were labeled with fluorochrome-conjugated monoclonal antibodies (see Table 2) for 30 min at room temperature, protected from light. After incubation, cells were washed with PBS and resuspended in 300 µL of PBS containing 1% paraformaldehyde (PFA) for fixation. Samples were then stored at 4 °C in the dark until flow cytometric analysis. Flow cytometric measurements were performed using a BD FACS Canto II flow cytometer (BD Immunocytometry Systems, Erembodegem, Belgium), and data acquisition was carried out using BD FACS Diva V6 software. Flow cytometric data were analyzed using FCS Express version 4.

**Figure 8:**
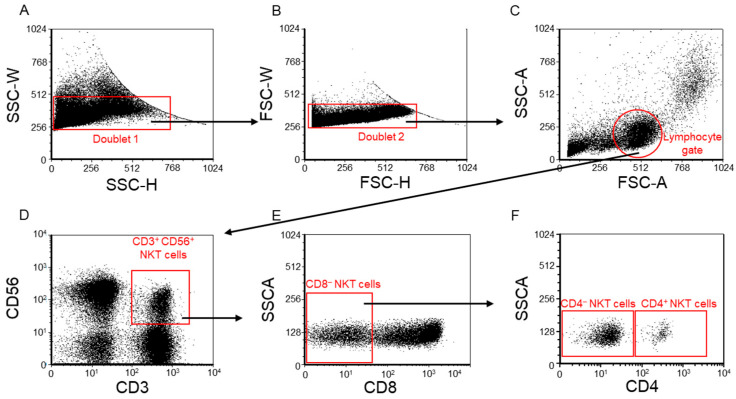
**Flow cytometric identification of CD4^+^ and CD4^−^ of NKT subpopulations.** The flow cytometric gating strategy to separate the examined CD4^+^ and CD4^−^ NKT subpopulations. After a doublet exclusion step (**A**,**B**) using FSC-A/SSC-A parameters, the lymphocyte gate was created (**C**). From the lymphocyte gate, the NKT cell population was gated based on the CD3^+^/CD56^+^ combination (**D**). From the NKT subpopulations, the CD8^−^ NKT subset was gated (**E**). From the CD8^−^ NKT cell subsets, CD4^−^ NKT and CD4^+^ NKT subpopulations were separated (**F**).

After thawing, events were filtered to singlets and lymphocytes by FSC/SSC, followed by identification of NKT cells as CD3^+^CD56^+^ (Figure 8). From this gate, CD8^−^ events were selected, and two final phenotypes were defined and analyzed throughout: (1) CD4^+^ NKT: CD3^+^CD56^+^CD8^−^CD4^+^; (2) CD4^−^ (double-negative) NKT: CD3^+^CD56^+^CD8^−^CD4^−^ (Figure 8F).

### 4.5. Statistical Analysis

To investigate the effect of the surface receptor CD4 and the impact of the different trimesters on the expression pattern of immune checkpoint molecules in NKT cells, a two-way ANOVA was applied for statistical significance testing in R, version 4.4.1. Explanatory variables were log-transformed before analysis. Decisions on the transformation of variables depended on visual inspection of “model-checking plots” in R for the models with transformed vs. untransformed variables. These plots allow for assumptions about the normality of residuals and variance homogeneity to be checked. For pairwise comparisons, Tukey post hoc tests were conducted to compare each cell type/trimester combination to another.

## Figures and Tables

**Table 1:** Gynecological and demographic data of the participating women.

	Non-Pregnant	1st Trimester	2nd Trimester	3rd Trimester
**No. of women**	20	26	28	29
**Age (years)**	29.85 (22–43)	32.77 (18–41)	32.54 (18–43)	32.10 (23–44)
**Gestation age at sampling (weeks)**	-	13.96 ± 2.44	24.96 ± 1.82	30.86 ± 3.77
**Gestation age at birth (weeks)**	-	39.05 ± 1.39	38.65 ± 1.87	39.34 ± 0.81
**Gravidity**	-	0.76	0.77	0.89
**Parity**	-	1.15	1.08	1.41

Statistical comparisons were made in R using one-way ANOVA tests. Data are shown as the mean value ± standard deviation (SD) of the mean. Statistical differences among the investigated cohorts were not detected.

**Table 2:** Fluorochrome-conjugated monoclonal antibodies used in the study.

Antigen	Format	Clone	Isotype	Manufacturer	Catalog No
CD3	BV510	UCHT1	Mouse BALB/c IgG1, κ	BD Biosciences	563109
CD4	FITC	RPA-T4	Mouse IgG1, κ	BD Biosciences	555346
CD8	APC-H7	SK1	Mouse BALB/c IgG1, κ	BD Biosciences	560179
CD226	BV421	DX11	Mouse BALB/c IgG1, κ	BD Biosciences	742493
TIGIT	PE	A1553G	Mouse IgG2a, κ	Biolegend	372704
CD112	PE	R2.525	Mouse IgG1, κ	BD Biosciences	551057
CD155	APC	SKII.4	Mouse IgG1, κ	Biolegend	337618
PD-1	PE	PD1.3	IgG2b Mouse	Beckmann Coult	B30634
PD-L1	BV421	MIH1	Mouse BALB/c IgG1, κ	BD Biosciences	563738
LAG-3	PerCp	11C3C65	Mouse IgG1, κ	Biolegend	369312
Galectin-3	PE	B2C10	Mouse BALB/c IgG1, κ	BD Biosciences	565676
NKG2D	PE-Cy7	1D11	Mouse RBF/DnJ IgG1, κ	BD Biosciences	562365

## Data Availability

The data used to support the findings of this study are available from the corresponding author upon request.
